# The Effects of Criminal Punishment on Medical Practices in the Medical Environment

**DOI:** 10.3390/ijerph16040604

**Published:** 2019-02-19

**Authors:** Munjae Lee

**Affiliations:** Department of Medical Device Management and Research, SAIHST, Sungkyunkwan University, Seoul 06351, Korea; emunjae@skku.edu

**Keywords:** doctors, medical disputes, overtreatment, medical practice, Korea Medical Dispute Mediation and Arbitration Agency, Korea

## Abstract

Recently, there have been cases in which doctors were criminally convicted for misdiagnosing a patient with constipation who then died of diaphragmatic hernia. The criminal punishment of doctors could create a side effect of reduced medical practitioners. This study analyzed the impact of medical disputes and deduced a plan to create a stable composition of the medical environment. An online survey was conducted with 79,022 doctors who are members of the Korea Medical Association. A total of 3109 responses were obtained, and the analysis used the questionnaire system of the Doctor’s News online survey system. The results demonstrated that doctors have become more psychologically inclined to prescribe overtreatments, avoidance treatments, and defensive treatments. Also, it was found that specialized agencies for medical appraisals were necessary. In order to resolve medical disputes objectively, it is necessary to improve credibility by securing the expertise of the Korea Medical Dispute Mediation and Arbitration Agency. In addition, there is a need for specialized agencies to undertake the medical appraisals and training of medical staff to build up their understanding of medical disputes. Thus, medical disputes can be minimized, and the fairness of medical dispute results can be strengthened.

## 1. Introduction

Most doctors face at least one case of malpractice across their career, and it often leads to costly and unresolved medical disputes. When medical disputes interfere with the time and specialization of doctors, it is referred to as a defensive treatment [1]. If treatment is the cause of a patient’s injury or death, this may lead to a lawsuit being filed against the medical practitioner [2]. Furthermore, the number of medical disputes has rapidly increased as the trust between doctors and patients has become fraught in a system of low insurance, low bills, and low income, which leads to such situations as the three-minute consultation and the overall destruction of the healthcare delivery system.

Due to characteristics such as the specialization of medicines, the specificity of medical practices, and the reticence of medical practitioners, it is difficult to recover the damages created by medical disputes. In any medical dispute, the patient must prove that the harm that resulted from the medical practice was malpractice and has to burden the responsibility of proving the illegality of the harm done. However, because patient conditions are variable and serious illness can be exacerbated naturally, it is not easy for experts to demonstrate the responsibility of a medical practitioner in the consequences of their medical practice. In addition, some rare medical conditions (e.g. heavy metal poisoning or autoimmune disorder) present with atypical symptoms and require a long time to reach an accurate diagnosis with wrong treatment being initiated [3,4]. However, the social and financial costs incurred by both patients and medical practitioners in order to resolve disputes are rapidly increasing as the number of medical disputes increase, and the duration needed for a resolution to be achieved is extended [5]. In addition, there is the phenomenon of doctors who intentionally perform defensive treatments or overtreatments due to the risk of medical accidents and who avoid medical fields that experience a high frequency of accidents. The Korea Medical Dispute Mediation and Arbitration Board was thus established to resolve medical conflicts quickly, fairly, and efficiently. This changed how disputes are resolved to more autonomous means, such as the practice of mediation or arbitration between the disputes with the help of experts such as medical professionals and legal experts. It thus became possible to resolve the conflicting relationships between disputers in better ways and maintain smoother relationships [6,7]. 

However, there was a recent incident in which three doctors in Seongnam City, South Korea, were sentenced to prison and held in custody for failing to properly treat a diaphragmatic hernia in a patient due to their misdiagnosis of the symptoms as constipation. This was a ruling that did not take into account the specific nature of medical practice. If the results of medical practice can lead to criminal punishment, the upshot will be that doctors only perform defensive, passive, and avoidant medicine [8]. This would ultimately lead to a greater burden being placed on patients as well as an increase in national medical costs. The Mediation Arbitration Board contributed to the medical review in the civil dispute that had convened prior to the three doctors being taken into custody. However, the medical review of the Mediation Arbitration Board was performed by a single doctor. This demonstrated a lack of subjectivity, and the fairness of this judgment seems questionable. Our study, therefore, analyzes the effect that a medical dispute may have on medical practice and proposes suggestions for the stable development of medical environments. 

## 2. Materials and Method

This research identifies the effect that the imprisonment of doctors has on medical practice and proposes suggestions for the stable development of medical environments. For this purpose, I conducted a nationwide survey of doctors in South Korea. My investigation occurred between November 3 and 6, 2018. The surveys were conducted online using the email addresses of 79,022 doctors who are members of the Korean Medical Association. A total of 3109 doctors participated in the survey. The analysis was performed using the Doctor’s News online survey system [9]. 

Examining the demographical characteristics of the survey respondents, most participants were men. Among the 3109 respondents, 2420 were male (80.2%) and 599 were female (19.8%). The age groups were categorized as doctors in their 40s (1112 respondents, 36.8%), doctors in their 50s (852 respondents, 28.2%), doctors younger than 30 (657 respondents, 21.8%), and doctors older than 60 (398 respondents, 13.2%), and the majority of respondents were in their 40s and 50s. The main work locations of the doctors were Seoul (866 respondents, 28.7%), Gyeonggi (500 respondents, 16.6%), and Busan (442 respondents, 14.6%), which demonstrates that the doctors are concentrated in the metropolitan area of Seoul and other major cities. The occupations of the doctors included private doctors (1230 respondents, 40.7%), salaried doctors (909 respondents, 30.1%), and professors (649 respondents, 21.5%) (Table 1).

Analyzing the results of the medical fields of the survey respondents, the most common practices were internal medicine (570 respondents, 18.9%), family medicine (233 respondents, 7.7%), emergency medicine (228 respondents, 7.6%), surgery (212 respondents, 7.0%), and gynecology (178 respondents, 5.9%). In particular, the interest of doctors in fields related to the imprisonment incident was high. The proportion of emergency medicine doctors in the survey was 7.6% (228 respondents) compared to 1.6% for specialty doctors among the Korean Medical Association, demonstrating a response rate approximately five times higher than other response rates (Table 2).

## 3. Results

The surveys were conducted with the intention of analyzing the effect that the imprisonment of the three doctors had on medical practice. First, we analyzed the perception of doctors on the incident and its resultant side effects. The results are as follows (Table 3). 

Most respondents (2868 respondents, 95%) responded that the court decision to imprison the three doctors for their role in causing the unfavorable results by missing the treatment period due to misdiagnosis was unfair, and viewed the criminal punishment of medical practice from goodwill as “unfair.” Furthermore, in considering whether the burden of doing additional tests on patients who demonstrate abnormal results, even if unnecessary, has become large as a result of this court decision, 97.3% of the respondents thought this to be “very true” (2588 respondents, 85.7%) or “slightly true” (350 respondents, 11.6%). Therefore, the mentality of performing unnecessary additional tests has increased due to this decision, and it seems that this will lead to doctors practicing overtreatment. The majority of respondents (2654 respondents, 87.9%) indicated that there was now a higher tendency to send a patient to a tertiary hospital if they demonstrate abnormal results, which demonstrates that doctors are now more likely to choose avoidant medicine (i.e., transferring patients to tertiary hospitals). To the question “has active medical practice become constrictive after this decision?”, responses of “very true” (2243 respondents, 74.3%) and “slightly true” (601 respondents, 19.9%) were high. More than nine out of ten doctors said that they would perform passive medical practice. 

I then analyzed the medical disputes that doctors experience in real medical situations and resolutions. The results are as follows (Table 4). 

To the question of *whether they had experienced a medical dispute in the past three years*, 31.9% (969 respondents) responded that they had, demonstrating that one out of three doctors experiences medical disputes. Therefore, doctors who have experienced medical disputes at least once seem to feel empathy for the three imprisoned doctors and fear that this could happen to them. Furthermore, the *type of resolution preferred by doctors who have experienced medical disputes or have the possibility of experiencing them in the future* was in the order of an out-of-court settlement (1186 respondents, 39.3%), the Korea Medical Association Medical Indemnity Mutual Insurance Association (1066 respondents, 35.3%), the Korea Medical Dispute Mediation and Arbitration Board (389 respondents, 12.9%), civil disputes (251 respondents, 8.3%), and others (118 respondents, 3.9%). By this evidence, it was demonstrated that the trust that doctors have for the Korea Medical Dispute Mediation and Arbitration Board, a public institution for which legal authority has been entrusted according to the Medical Disputes Mediation Act (Act of Medical Malpractice Damages Relief and Mediation for Medical Dispute Resolution) is low. On the question of whether the Korea Medical Dispute Mediation and Arbitration Board was responsible for medical reviews that can be the basis of criminal punishment, responses indicating that the Mediation Arbitration Board’s role in medical reviews was “very undesirable” (1670 respondents, 55.3%) and “not very desirable” (830 respondents, 27.5%) were high, with a total of 82.8% negatively viewing the Mediation Arbitration Board’s role in medical reviews. 

Finally, I analyzed the survey with regards to the resolutions and plans for the punishment of medical practitioners due to occupational negligence. The results are as follows (Table 5). 

From the respondents, 96.3% of the doctors said that the enactment of a special law on medical accidents was necessary, with 2240 respondents (74.2%) responding “very desirable” and 667 respondents (22.1%) responding “desirable” to the *plan of enacting a special law on medical accidents to resolve medical practice with non-criminal payments (recovery of damage) rather than criminal punishment*. Furthermore, 98.7% of the doctors thought that education on medical disputes was necessary, with 2642 respondents (87.5%) responding “very necessary” and 338 respondents (11.2%) responding “slightly necessary” to *the necessity of education for doctors or medical professionals who are preparing for medical disputes*. Currently, the court is also conducting a medical review in the event of a medical dispute. To this, 95.3% of the doctors who responded agreed with the necessity of a medical case review institution, with 2476 respondents (82%) responding “very necessary” and 402 respondents (13.3%) responding “slightly necessary” to *the need for an institution for professional medical reviews.*

## 4. Discussion

In this research paper, We have analyzed the effect that the recent imprisonment of three doctors had on medical practice. In the section below, We put forward proposals that can develop a more stable medical environment. 

First, the objectivity of the Korea Medical Dispute Mediation and Arbitration Board must be secured in order to strengthen its functions. In South Korea, the “Act of Medical Malpractice Damages Relief and Mediation for Medical Dispute Resolution” (hereafter referred to as the Medical Disputes Mediation Act) was enacted in 2011 and has been in effect since April 2012. After the implementation of the Medical Disputes Mediation Act, the Korea Medical Dispute Mediation and Arbitration Board was established as an independent mediation institution for medical disputes [10]. Additionally, the medical accident review board was established within the Mediation Arbitration Board to investigate the facts required for the arbitration or mediation of medical disputes and identify the existence and causal relationships surrounding medical practice [11]. The Mediation Arbitration Board is comprised of the Mediation Arbitration Committee, which has between 50 and 100 non-permanent Mediation Members, each of whom have a certain level of qualification. In order to perform the function of the Mediation Arbitration Committee efficiently, there are departments by field, subject, or region that are comprised of five Mediation Members. The department investigates the causal relationship of the medical accident and malpractice and makes arbitration judgments by computing the compensation for damages. For Mediation Members, there are two medical practitioners, two law experts, and one consumer rights member. However, 60% of the review members are non-medical professionals, and due to the speed at which the reviews are completed, reliability problems arise [12]. Most of the victims of medical accidents and their families have said that the Mediation Arbitration Board is more focused on a settlement with the hospital than it is with arbitrating the medical accident.In contrast, the medical field has the view that objectivity is lacking in the Mediation Arbitration Board, with cases of transfers due to underlying diseases, and complications being exacerbated by other medical institutions being included in the malpractice claims for which the Mediation Arbitration Board enforces their mediation procedures [13]. It seems that the Korea Medical Dispute Mediation and Arbitration Board cannot perform its role effectively. Therefore, patients or medical practitioners must mediate or arbitrate between themselves based on the review results to which they cannot medically or objectively dissent. The review results of the Mediation Arbitration Board can be submitted as evidence in a future lawsuit, so the accuracy of the medical judgment is very important. Therefore, the review board that exists within the Mediation Arbitration Board must be separate so that judgment on the medical practice and the legal issue must be made by separate entities. Furthermore, in the composition of the review board, a legal enactment that both expands the participation of medical professionals and strengthens the transparency of the composition is necessary. Through this, the objectivity and specialization of the Korea Medical Dispute Mediation and Arbitration Board can be obtained to increase satisfaction with and reliability of the patients and medical institutions. 

Second, a special act for resolving medical accidents with non-criminal payments must be enacted. The political trial of the judiciary induces defensive treatment practices and has a negative impact on the health of citizens [14]. One representative phenomenon of defensive treatment is overtreatment. Overtreatment refers to medical care in which the number of tests is increased to avoid the responsibility of not having performed early medical care due to not having done follow-up tests or imaging. Due to the recent imprisonment of doctors, there has been an increase in doctors who perform overtreatment, and this leads to an increase in the costs of medical resources and finances. In contrast, the results of medical practice leading to criminal punishment, regardless of the best possible medical care, can lead to avoidant medicine or minimal medicine [15]. Therefore, the viewpoint of medical practitioners is that the enactment of a special act on medical accidents is necessary. An act such as this can recover the damages made to the patients and provide monetary compensations through non-criminal payments. This is preferable to the enforcement of criminal punishment when medical accidents occur. A special act on medical accidents will exempt criminal punishment except in cases of medical negligence or when the malpractice is done on purpose. The medical practice of doctors is inherently well-intentioned, and it seems unfair to be punished with criminal punishment for bad results even though the best possible treatment was given. Therefore, the enactment of a special act on medical accidents is necessary to build a medical environment in which doctors and citizens are safe by resolving the anxiety of unavoidable situations that occur in medical scenarios. Furthermore, the concept of a special act seems to be the basis for reasonable resolutions of medical disputes between patients and medical practitioners. 

Third, a specialized institution for medical reviews and the continuing education of medical professionals in preparation for medical disputes is necessary. In the Korea Medical Dispute Mediation and Arbitration Board, there are currently some education programs that teach an understanding of “The Act of Medical Malpractice Damages Relief and Mediation for Medical Dispute Resolution,” as well as mediation and arbitration procedures, the introduction to cases, and training on plans for the prevention of medical accidents. However, the training is only about one hour and is only scheduled for twice a day. This demonstrates that systematic education is a difficult procedure. Therefore, ensuring the continuing education of practitioners is necessary, and it should be enacted by expanding the education program in the Mediation Arbitration Board as well as the length of training. Opening education streams that strengthen communication lines with patients, such as training on communication, ethics, and personality training, can help to solve conflicts between patients and doctors. In this way, medical disputes may ultimately be reduced [16]. Moreover, education for the reviewers is also necessary. Currently, in the laws related to medicine, there is no inclusion of a specialized certification procedure, a requirement for reviewers, or specialized rules for the qualifications of medical professionals who act as reviewers except in civil litigation laws. Physical reviews, a legal procedure, can be performed with a reasonable review using medical and law-related knowledge. Moreover, the results of physical reviews may differ between hospitals or doctors, thus making it difficult to verify the reliability of the physical review. Therefore, a systematic training procedure is necessary for doctors who perform physical reviews. This will lead to the development of specialized institutions for medical reviews. Therefore, the establishment of a medical review institution that conducts its reviews with speed, fairness, and specialization and also fulfills the various medical review demands is also necessary. 

## 5. Conclusions

Medical disputes arise when the outcome of medical actions based on the trust between doctors and patients is considered negative. Although medical practitioners stress certain inevitable consequences due to the specific nature of medical practice, patients who cannot accept this will turn the medical accident into a medical dispute [11]. If the outcomes of these medical disputes lead to the criminal punishment of doctors, the public will suffer from damages such as the possibility that dangerous surgeries with high death rates or complicated treatments will be avoided. To analyze these problems, a survey was conducted on doctors, and the results are as follows. 

First, the specialization and reliability of the Korea Medical Dispute Mediation and Arbitration Board must be increased. For this purpose, specialization must be obtained by securing a reviewer with a medical practitioner background in each specialized field. Additionally, the composition of the review board and the review procedure itself must be transparent to verify its reliability. Second, a special act on medical accidents must be enacted to resolve medical disputes involving non-criminal payments. Therefore, the complete recuperation of damages should be carried out based on treatment or compensation rather than criminal punishment for unintentional medical practices. Third, a specialized body for the continuous education of medical disputes and medical appraisals should be established. For medical reviews, a review expert should conduct a neutral review that is not biased. Therefore, through the establishment of a specialized medical reviewer body, it will become necessary to cultivate review experts through systematic education to produce fair and objective reviews. 

Through this, the present study has intended to minimize medical disputes by improving the medical environment and strengthening the fairness and objectivity of the results of medical disputes. However, We are limited in that We could not analyze the effects on the perceptions of patients. This analysis was limited to the effect of the imprisonment of three doctors only from the point of view of other doctors. Future research will need to search for ways to address the differences in perception by comparing the changes in perceptions between doctors and patients. A sense of trust should be established between patients and doctors in medical practice, but this particular incident has highlighted the potential problems that can arise due to dissatisfaction among patients (and their family members) who have experienced medical accidents. In particular, patients reported needing sufficient time for doctors to explain the nature of diseases, and doctors also say that an increase in the duration of contact time with patients will lead to a decrease in medical accidents. Therefore, in order to effectively reduce medical disputes, reliability should be increased by increasing the contact time between patient and doctor through the activation of primary care and the improvement of the medical system. 

## Figures and Tables

**Table 1:** Demographic characteristics of the survey respondents.

		Frequency	%
Sex	Male	2420	80.2
	Female	599	19.8
Age	Younger than 30	657	21.8
	40s	1112	36.8
	50s	852	28.2
	Older than 60	398	13.2
Location	Seoul	866	28.7
	Gyeonggi	500	16.6
	Busan	442	14.6
	Daegu	178	5.9
	Gyeongam	118	3.9
	Incheon	116	3.8
	Gwangju	115	3.8
	Gyeongbuk	114	3.8
	Gangwon	105	3.5
	Chungnam	87	2.9
	Jeonbuk	88	2.9
	Chungbuk	82	2.7
	Daejeon	80	2.6
	Jeonnam	57	1.9
	Ulsan	44	1.5
	Jeju	27	0.9
Occupation	Private doctors	1230	40.7
	Salaried doctors	909	30.1
	Professors	649	21.5
	Other	85	2.8
	Training doctors	83	2.7
	Army surgeons	34	1.1
	Public doctors	29	1.0

**Table 2:** Analysis of the medical fields of the survey respondents.

		Frequency	%
Field	Internal medicine	570	18.9
	Family medicine	233	7.7
	Emergency medicine	228	7.6
	Surgery	212	7.0
	Gynecology	179	5.9
	Orthopedics	177	5.9
	Pediatrics	169	5.6
	Anesthesiology	133	4.4
	Neuropsychiatry	127	4.2
	Otorhinolaryngology	115	3.8
	Neurosurgery	109	3.6
	Ophthalmology	102	3.4
	Urology	88	2.9
	General physician	88	2.9
	Radiology	86	2.8
	Neurology	82	2.7
	Dermatology	74	2.5
	Rehabilitation medicine	72	2.4
	Plastic surgery	59	2.0
	Cardiothoracic Surgery	43	1.4
	Pathology	17	0.6
	Basic medicine	12	0.4
	Nuclear medicine	11	0.4
	Occupational medicine	10	0.3
	Radiation oncology	6	0.2

**Table 3:** Knock-on effects following the imprisonment incident.

	Frequency	%
*The court decision to imprison the three doctors for their role in causing the unfavorable results by missing the treatment period due to misdiagnosis was unfair.*		
**Unfair**	2868	95.0
**Fair**	100	3.3
**Don’t know**	51	1.7
*The burden of doing additional tests on patients who demonstrate abnormal results, even if unnecessary, is now greater.*		
**Very true**	2588	85.7
**Slightly true**	350	11.6
**No difference**	63	2.1
**False**	18	0.6
*The tendency to send a patient to a tertiary hospital if they demonstrate abnormal results is now greater.*		
**Very true**	2,654	87.9
**Slightly true**	302	10.0
**No difference**	60	2.0
**False**	3	0.1
*Has active medical practice become constrictive after this decision?*		
**Very true**	2243	74.3
**Slightly true**	601	19.9
**No difference**	103	3.4
**False**	72	2.4

**Table 4:** Possible resolutions for medical disputes.

	Frequency	%
*Whether doctors have experienced a medical dispute in the past three years.*		
**Yes**	963	31.9
**No**	2056	68.1
*The resolution preferred by doctors who have experienced medical disputes or have the possibility of experiencing them in the future.*		
**An order of out-of-court settlement**	1186	39.3
**The Korea Medical Association Medical Indemnity Mutual**	1066	35.3
**The Korea Medical Dispute Mediation and Arbitration Board**	389	12.9
**Civil disputes**	251	8.3
**Others**	118	3.9
**The Korea Consumer Agency**	9	0.3
*The role of the Mediation Arbitration Board in medical reviews.*		
**Very undesirable**	1670	55.3
**Not very desirable**	830	27.5
**Desirable**	151	5.0
**Very desirable**	24	0.8
**Don’t know**	344	11.4

**Table 5:** The necessity of education and an institution for medical disputes.

	Frequency	%
*The plan of enacting a special law on medical accidents to resolve medical practice with non-criminal payments (recovery of damage) rather than criminal punishment.*		
**Very desirable**	2240	74.2
**Desirable**	667	22.1
**Not very desirable**	24	0.8
**Don’t know**	88	2.9
*The necessity of education for doctors or medical professionals who are preparing for medical disputes.*		
**Very necessary**	2642	87.5
**Slightly necessary**	338	11.2
**Slightly unnecessary**	18	0.6
**Very unnecessary**	9	0.3
**Don’t know**	12	0.4
*The need for an institution that undertakes professional medical reviews.*		
**Very necessary**	2476	82.0
**Slightly necessary**	402	13.3
**Slightly unnecessary**	69	2.3
**Very unnecessary**	24	0.8
**Don’t know**	48	1.6
